# C1q nephropathy- unity in diversity

**DOI:** 10.12861/jrip.2013.37

**Published:** 2013-10-10

**Authors:** Pavan Malleshappa

**Affiliations:** ^1^Division of Nephrology, Department of Medicine, Adichunchanagiri Institute of Medical Sciences, B. G. nagara, Karnataka state, India

**Keywords:** C1q nephropathy, Minimal change disease, Immunofluorescence

**Implication for health policy/practice/research/medical education:**

C1q nephropathy refers to a disorder in which C1q deposits are seen in mesangium on immunofluorescence microscopy and mesangial electron dense deposits on electron microscopy. The diagnosis of C1q nephropathy is based on demonstration of intense C1q (dominant or co-dominant) positivity, mainly in the mesangium on immunofluorescence microscopy. Electron dense deposits of C1q in C1q nephropathy are confirmatory of the diagnostic entity.

C1q nephropathy refers to a disorder in which C1q deposits are seen in mesangium on immunofluorescence microscopy and mesangial electron dense deposits on electron microscopy (1). C1q nephropathy was first proposed by Jennette and Hipp as a distinct clinical entity that caused glomerulonephritis in the absence of SLE (2), with dominant or co-dominant deposition of C1q in the mesangial area.

C1q is the first component of the classical pathway of complement activation (3). C1q is a 400 kDa protein. C1q comprises 6 A, 6 B and 6 C chains, each possessing a globular N-terminal domain and a conserved C-terminal region. C1q activates the classical pathway by attaching to the Fc portion of IgM and some IgG subtypes after they have bound antigen. This theory suggests that complement activation by antigen-antibody complexes in the glomeruli is the reason for pathogenesis of C1q nephropathy (3). But the alternative and lectin pathways are also involved in C1q nephropathy (4). The exact pathogenesis leading to mesangial C1q deposition and the mechanism by which C1q deposition is likely to cause disease has remained enigmatic.

Earlier reports on C1q nephropathy had specific histopathological patterns of mainly focal or diffuse mesangial proliferative glomerulonephritis. Several patterns have subsequently been reported. The histologic patterns of C1q nephropathy could be divided broadly as: a) minimal change disease (MCD), b) focal segmental glomerulosclerosis and c) immune mediated proliferative glomerulonephritis (GN) (5). Later group encompasses different morphologic appearances ranging from focal/diffuse mesangial proliferative GN, post infectious GN, membranoproliferative GN, and membranous GN. Occasional case report of crescentic glomerulonephritis, tubulointerstitial nephritis, and thin basement membrane nephropathy is also seen in C1q nephropathy. Irrespective of light microscopic wide spectrum, the diagnosis of C1q nephropathy is based on demonstration of intense C1q (dominant or co-dominant) positivity, mainly in the mesangium on immunofluorescence microscopy. Electron dense deposits of C1q in C1q nephropathy are confirmatory of the diagnostic entity. C1q deposits are always seen in mesangium, deposits can also be seen in other areas depending on morphologic appearances on light microscopy.

The prevalence of C1q nephropathy ranges from 0.2 to 6% in renal biopsies. Clinical diagnosis remains controversial because of its heterogeneity. Typically, C1q nephropathy is characterized by onset in older children and young adults with severe proteinuria or nephrotic syndrome, with resistance to steroid treatment, frequent recurrence, and a poor long-term prognosis. C1q nephropathy may also present as nephritic syndrome or isolated proteinuria/hematuria. Hypertension and renal insufficiency at the time of diagnosis is quite frequent. Primary C1q nephropathy is divided into two variants, such as MCD/FSGS variant and immune-complex GN variant. Immune complex GN variant includes mesangial proliferative glomerulonephritis, membranous nephropathy and membranoproliferative- like glomerulonephritis. Secondary C1q nephropathy may be seen in patients with viral infections, diabetes (6) and rarely with rheumatoid arthritis.

The treatment of C1q nephropathy remains a clinical challenge because the available evidence base is limited in scope. Current therapy involves treatment of the underlying light microscopic lesion. Glucocorticoids remain the mainstay of treatment but most of the studies have indicated poor response to therapy (7). Methylprednisolone pulse therapy has shown to be effective in steroid resistant cases. Therapy with cyclophosphamide, azathioprine, mycophenolate mofetil, tacrolimus and rituximab used separately or in combination with steroids has shown some clinical response in different studies. Unfortunately, there are no published prospective, controlled studies that have examined efficacy of therapy in C1q nephropathy. Much work is needed to better understand the pathophysiology, which may allow identification and development of more specific therapy options in the future.

## Author’s contribution

PM is the single author of the manuscript.

## Conflict of interests

The author declared no competing interests.

## Ethical considerations

Ethical issues (including plagiarism, misconduct, data fabrication, falsification, double publication or submission, redundancy) have been completely observed by the authors.

## Funding/Support

None.
